# Comparative Effectiveness of Artificial Intelligence Versus Conventional Methods for Detecting Peritoneal Metastasis in Colorectal Cancer: A Systematic Review

**DOI:** 10.7759/cureus.95484

**Published:** 2025-10-27

**Authors:** Mohamed Elsaigh, Safa Baqar, Bakhtawar Awan, Omnia S Saleh, Mohamed Hesham Gamal, Naomi Abara, Alexander Hawkins, Besiana P Beqo

**Affiliations:** 1 Emergency Surgery, Northwick Park Hospital, London North West University Healthcare NHS Trust, London, GBR; 2 General Surgery, Royal United Hospitals Bath NHS Foundation Trust, Bath, GBR; 3 General Surgery, Northwick Park Hospital, London North West University Healthcare NHS Trust, London, GBR; 4 Surgery, Brigham and Women's Hospital, Boston, USA; 5 Pharmacology and Therapeutics, Faculty of Pharmacy, Tanta University, Tanta, EGY; 6 General Surgery, Royal Cornwall Hospitals NHS Trust, Truro, GBR; 7 General Surgery, Royal Cornwall Hospital, Truro, GBR; 8 General Surgery, Boston Children’s Hospital, Harvard Medical School, Boston, USA

**Keywords:** artificial intelligence, colorectal cancer, deep learning, machine learning, peritoneal metastasis, predictive modeling, radiomics, systematic review

## Abstract

Colorectal cancer represents a major global malignancy and a leading cause of cancer-related death. Peritoneal metastasis occurs in a significant proportion of colorectal cancer patients and is associated with markedly worse prognosis compared to other metastatic sites, with a limited median overall survival. Early detection remains challenging due to the limited sensitivity of conventional imaging techniques, with computed tomography exhibiting poor detection rates for small lesions and necessitating invasive diagnostic procedures for accurate diagnosis. The limitations of traditional diagnostic modalities have driven a growing interest in artificial intelligence applications to advance the early, non-invasive detection of peritoneal metastasis. This study aimed to systematically assess whether artificial intelligence and machine learning approaches enhance the accuracy and efficiency of detecting peritoneal metastasis and predicting tumor spread patterns compared to conventional imaging and clinical assessment methods in patients with colorectal cancer. A systematic review was conducted in accordance with Preferred Reporting Items for Systematic Reviews and Meta-Analyses (PRISMA) guidelines, searching the PubMed, Web of Science, Cochrane, Embase, and Scopus databases for studies published between 2015 and 2025. The search strategy included comprehensive terminology related to artificial intelligence and machine learning, combined with terms related to peritoneal metastasis. Two independent reviewers assessed study quality using the Quality Assessment of Diagnostic Accuracy Studies-2 (QUADAS-2) tool for diagnostic accuracy studies and the modified Radiomics Quality Score for artificial intelligence (AI) and radiomics studies, with disagreements resolved through consensus discussions.

From multiple countries, 22 studies were included with a total population of over 40,000 patients. AI applications consistently outperformed traditional methods across all modalities. While conventional approaches showed moderate performance with C-indices of 0.73-0.85 and CT imaging missed 89% of small lesions, AI-assisted systems demonstrated superior results as follows: cytological detection achieved over 95% accuracy and 99% specificity; radiomics models reached AUCs up to 0.941; circulating tumor DNA integration provided 8.5-fold increased risk identification; and computer-assisted staging laparoscopy improved surgical diagnostic accuracy from 52% to 79% compared to human assessment alone. AI technologies demonstrate promising advantages for peritoneal metastasis detection, offering enhanced diagnostic accuracy, objective assessments, faster analysis, and improved clinical decision-making, particularly through human-AI collaboration. However, most studies lack external validation across diverse populations and real-world settings, while current implementations face significant workflow challenges. Before clinical adoption, future research must prioritize large-scale prospective validation studies, external validation across diverse populations, and comprehensive cost-effectiveness analyses to ensure safe and effective integration into clinical practice.

## Introduction and background

Colorectal cancer (CRC) is the third most frequently diagnosed malignancy worldwide and the second leading cause of cancer-related death [1]. According to global cancer statistics, over 1.9 million new cases of CRC and approximately 935,000 associated deaths were reported in 2020 [1]. Metastasis is a primary cause of mortality in CRC, with the liver being the most common site of distant spread. The peritoneum is the second most prevalent site of metastasis, occurring in approximately 10 to 25% of CRC patients following initial diagnosis [2]. Clinically, peritoneal metastasis (PM) represents a particularly aggressive form of disease progression and is associated with markedly worse progression-free and overall survival compared to other metastatic sites [3,4].

Over the past two decades, peritoneal metastasis from colorectal cancer (PMCC) has been considered a locally progressive disease. This oversight has driven efforts to develop and refine palliative treatment strategies targeting the peritoneal cavity. Despite these advancements and the application of aggressive medical interventions, the median overall survival for patients with PMCC remains limited, ranging from 10 to 18 months [5]. CRC is not the only source of PM, as it has also been observed in ovarian tumors and other gastrointestinal tract (GIT) malignancies, such as gastric, hepatocellular, and pancreatic malignancies [6]. PM is the most prevalent site of distant metastasis in gastric cancer. Recent population-based studies demonstrated that 10-21% of gastric cancer patients suffer PM [7].

Even with advancements in systemic therapies and surgery, the outlook for patients with CRC-associated peritoneal malignancy is still bleak, highlighting the urgent need for better approaches in early detection, treatment, and monitoring. Early detection remains challenging due to the limited sensitivity of current radiological methods and the delayed clinical presentation. Conventional imaging techniques, such as computed tomography (CT), lack the necessary spatial resolution to identify initial peritoneal disease. CT has an overall sensitivity of approximately 43% for PM, with a detection rate of 94% for lesions larger than 5 mm; however, this drops to just 11% for those smaller than 5 mm [8]. Although positron emission tomography-computed tomography (PET-CT) has enhanced sensitivity (85%) and specificity (88%) [9].

Nowadays, direct surgical exploration through diagnostic laparoscopy or laparotomy remains the gold standard for a precise diagnosis. However, these techniques offer heightened sensitivity; they are invasive, expensive, and carry significant procedural risks. So, they are recommended only for high-risk patients [10]. That’s why it's essential to develop an accurate, sensitive, and non-invasive method for the early diagnosis of PM.

Artificial intelligence (AI) refers to computer systems designed to perform tasks that normally require human intelligence, such as recognizing patterns and making predictions. Machine learning (ML), a subset of AI, enables these systems to learn from data and improve their performance over time without being explicitly programmed [11-13]. Due to the limitations of conventional diagnostic modalities, there is a growing interest in using AI to advance the early, non-invasive detection of PM [11]. AI, particularly ML and deep learning algorithms, has demonstrated significant results in improving diagnostic precision through the analysis of complex, high-dimensional datasets derived from radiological imaging, histopathological slides, and clinical parameters [12,13].

A recent study has demonstrated that AI-based models, such as an 18F‑fluorodeoxyglucose (FDG) PET/CT radiomics‑based multimodality fusion model (MMF), can achieve superior predictive performance for PM compared to traditional clinical tools and expert assessment in cases of advanced gastric cancers, even in challenging histological subtypes like mucinous adenocarcinoma and signet ring cell carcinoma [14]. Another recent study showed that CT-based radiomics, particularly features derived from the tumor mesenteric fat space, can effectively predict occult peritoneal metastasis (OPM) in patients with advanced gastric cancer. A machine learning-based radiomics nomogram integrating multiple regions of interest achieved high diagnostic performance, with an area under the curve (AUC) up to 0.943 in the training set and 0.835 in the test set, highlighting its potential as a non-invasive tool for early metastasis detection [15].

Our primary objective was to systematically assess whether AI and ML approaches improve the accuracy and efficiency of detecting PM and predicting tumor spread patterns compared to conventional imaging and clinical assessment methods.

## Review

Methods

This review was conducted following the standards of the Preferred Reporting Items for Systematic Reviews and Meta-Analyses (PRISMA) guidelines [16]. This research aimed to confirm whether AI and machine learning (ML) approaches can enhance the accuracy and efficiency of detecting peritoneal metastasis, predicting tumor spread patterns, and determining the need for surgery compared to conventional imaging and clinical assessment methods.

Literature Search

We performed our search based on five different electronic databases (PubMed, Web of Science, Scopus, Cochrane, and Embase) using the search strategy of ("artificial intelligence" OR "computer intelligence" OR "computational intelligence" OR "machine intelligence" OR "deep learning" OR "deep neural network" OR "machine learning" OR "intelligent support" OR "intelligent agent" OR "neural network" OR "artificial neural network" OR "natural language processing" OR nlp OR "decision tree" OR "support vector machine" OR "reinforcement learning" OR "supervised learning" OR "unsupervised learning" OR "random forest" OR "naive bayes" OR "k-means" OR "convolutional neural network" OR "gradient boosting" OR "k-nearest neighbor" OR "Bayesian network" OR "principal component analysis" OR "apriori algorithm" OR "Long-short term memory" OR lstm OR "classification model" OR "least absolute shrinkage and selection operator" OR lasso OR "back-propagation" OR "latent dirichlet allocation" OR "topic-modelling" OR "multi-layer perceptron" OR "gated recurrent unit" OR "max-pooling" OR "temporal difference learning" OR "bag-of-words" OR "language model" OR "word embedding" OR "gaussian process" OR "computer vision" OR "fuzzy logic" OR "expert system*" OR "prediction model*" OR "predictive model*" OR "predictive algorithm*" OR "prediction algorithm*") AND (peritoneum OR peritoneal) AND (metastas*) with considering the research question to be answered. The complete search strategy for each database is presented in the appendix.

Inclusion Criteria

Only articles written in English were included, with the review scope defined as a 10-year period from 2015 to 2025. We included studies with human participants only, without specifying gender or age. We included studies conducted using patients with peritoneal metastasis that utilized an AI model, whether for detection, prediction, or imaging purposes.

Exclusion Criteria

Non-English publications were excluded, conference abstracts without full-text availability, duplicate publications or overlapping datasets, case reports and case series, studies conducted on animals, and articles with AI and gastric or epigastric metastasis (full detailed eligibility criteria are presented in Table 1).

**Table 1:** Eligibility criteria of the included studies.

Criteria	Inclusion	Exclusion
Publication period	2015-2025 (10-year period)	Publications outside the 2015-2025 timeframe
Study population	Human participants (all genders and ages)	Animal studies
Study design	Original research articles with full-text availability	Conference abstracts without full-text availability; case reports and case series
Medical condition	Studies involving patients with peritoneal metastasis	Studies focusing on gastric or epigastric metastasis
Technology focus	Studies utilizing artificial intelligence (AI) models for detection, prediction, or imaging purposes	Studies without an AI model application
Data integrity	Original datasets and publications	Duplicate publications or overlapping datasets

Critical Appraisal

In accordance with the eligibility criteria and PRISMA guidelines, the included articles were independently analyzed by two reviewers. Any reviewer discrepancies were resolved through discussions between the two authors until a consensus was reached, with the third author resolving any conflicts. Inter-reviewer agreement was assessed using Cohen's kappa statistic.

Quality Assessment

We employed two different quality assessment tools, tailored to the nature and design of each study. For diagnostic accuracy studies, we applied Quality Assessment of Diagnostic Accuracy Studies-2 (QUADAS-2), a well-established instrument specifically developed for evaluating such research in systematic reviews [17]. This tool examines potential bias across four key areas as follows: patient selection methods, the diagnostic test being evaluated, the reference standard used for comparison, and study flow and timing, along with three additional domains that assess the applicability of the findings to our research question [17].

For radiomics and AI studies, we employed a modified Radiomics Quality Score (RQS) assessment tool, which is specifically designed to evaluate the quality of radiomics research and AI model development [18]. Two independent reviewers scored each study using 16 standardized criteria (V1-V16) with points ranging from -3 to +36. The evaluation covered three main areas as follows: technical and methodological quality (V1-V4), statistical and validation approaches including feature reduction methods (V5-V12), and clinical translation potential including cost-effectiveness considerations (V13-V16). Studies were classified as high quality (≥20 points), moderate quality (15-19 points), or lower quality (<15 points) to help interpret their methodological strength and clinical applicability [18].

Results

**Figure 1:**
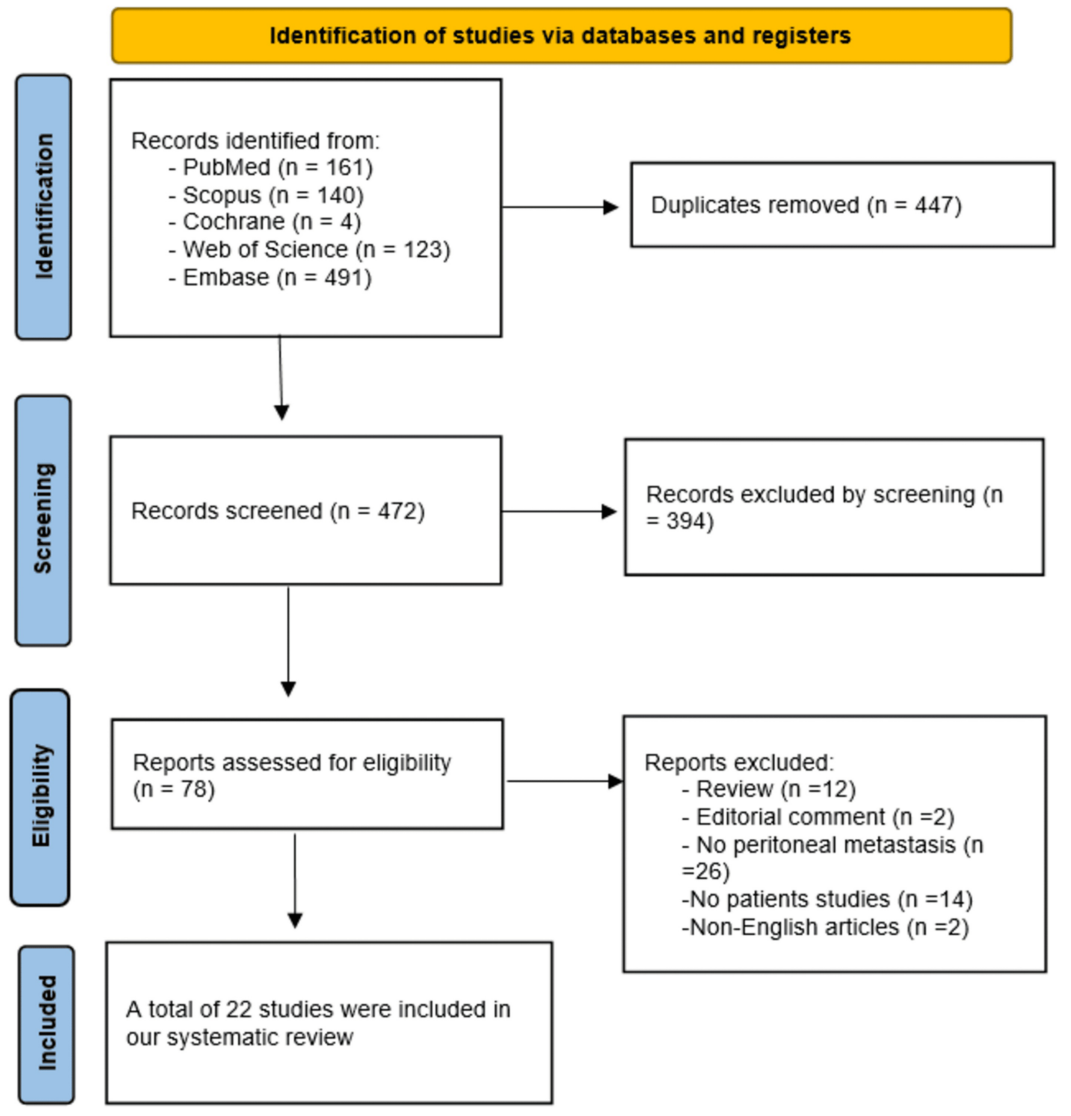
PRISMA flow diagram. PRISMA: Preferred Reporting Items for Systematic Reviews and Meta-Analyses

After performing our search strategy, a total of 919 results were obtained from the five libraries we searched. After excluding 447 duplications, we performed title and abstract screening, followed by full-text screening to ensure that we included only studies that matched our criteria. Finally, we included 22 studies that matched our criteria for the systematic review [19-40]. The flowchart of the strategy search for this systematic review has been summarized and presented in Figure 1.

Baseline and Summary of the Included Studies

The included studies were conducted across several countries, with China, Japan, South Korea, the United States, and France emerging as prominent contributors to research exploring AI applications in colorectal cancer with peritoneal metastasis. The majority of studies employed retrospective or cohort-based designs. A wide range of AI methodologies were applied, including traditional statistical approaches, such as least absolute shrinkage and selection operator (LASSO) and elastic net regression, as well as more advanced ML and deep learning techniques, notably predictive modeling algorithms and deep convolutional neural networks (DCNNs). These AI models were utilized for various clinical purposes, including the development of nomograms to predict metachronous peritoneal metastasis, the integration of circulating tumor DNA (ctDNA) data with clinical variables, and the support of cytological detection of metastatic cells in ascitic fluid. The tumor locations mainly encompassed the right and left colon and the broader colorectal area, while the site of metastasis was consistently the peritoneum. It is important to note that tumor (T) and lymph node (N) staging data were not uniformly reported or assessed across all patients. This may introduce variability in how tumor burden and nodal involvement were considered in each study. Comprehensive baseline and summary characteristics of the included studies are detailed in Table 2.

**Table 2:** Baseline characteristics and summary of the included studies. AI: artificial intelligence; AIIR: artificial intelligence iterative reconstruction; AJCC: American Joint Committee on Cancer; AUC: area under the curve; CASL: computer-assisted staging laparoscopy; CRC: colorectal cancer; CT: computed tomography; ctDNA: circulating tumor DNA; DCNN: deep convolutional neural network; DL: deep learning; DLR: deep learning-based radiomics; DPN: dual path network; HCC: hepatocellular carcinoma; HCR: handcrafted radiomics; HG: high grade; LASSO: Least Absolute Shrinkage and Selection Operator; mPC: metachronous peritoneal carcinomatosis; MSFF-Net: multi-scale feature fusion network: N: lymph node stage; NA: not available/not applicable; NLP: natural language processing; OPM: occult peritoneal metastases; OS: overall survival; PC: peritoneal carcinomatosis; PDAC: pancreatic ductal adenocarcinoma; PET/CT: positron emission tomography/computed tomography; PTB: peritoneal tuberculosis; SEER: Surveillance, Epidemiology, and End Results; SVM: support vector machine; T: tumor stage; TNM: tumor, node, metastasis staging system; USA: United States of America

Studies	Study design	Total patients	Country	Intervention	Tumor location	Location of metastasis	Type of AI used	T stage	N stage	Primary outcomes
Ban et al. (2023) [30]	Retrospective study	878	China	Predictive nomogram development	Colorectal (right, transverse, left colon/high rectum)	Peritoneal metastasis	LASSO regression for statistical modeling only	T1: 2.6%, T2: 6.4%, T3: 72.6%, and T4: 18.4%	N0: 43.8%, N1: 33.4%, and N2: 22.7%	Development of predictive nomogram for metachronous peritoneal metastasis in CRC
Fawaz et al. (2023) [29]	International retrospective cohort	235	Mainly France	Second-look laparoscopic exploration	Colon cancer	Peritoneal metastasis	Prediction model	T1-T2: 2.6%, T3: 43.4%, and T4: 54.0%	N0: 27.2%, N1: 40.9%, and N2: 31.9%	Prediction model for refining timing of early second-look laparoscopic exploration
Han et al. (2025) [27]	Retrospective study	299	China	Circulating tumor DNA (ctDNA) detection	Colorectal (right colon: 24.7%, left colon: 75.3%)	Peritoneal metastasis	Machine learning for predictive modeling only	T1: 0.9%, T2: 1.7%, T3: 43.4%, and T4: 54.0%	N0: 27.2%, N1: 40.9%, and N2: 31.9%	Predictive model combining ctDNA with clinical pathological risk factors for peritoneal metastasis
Kim et al. (2024) [28]	Retrospective analysis of a nationwide cytology dataset	581	South Korea	AI-assisted detection of metastatic cells in ascitic fluid	Colorectal	Ascitic fluid (peritoneal)	Deep convolutional neural network (DCNN) models including densenet161, DPN, inceptionresnetv2, inceptionv4, mobilenetv2, resnet152, resnext, senet154, Xception	NA	NA	Accuracy, sensitivity, and specificity for differentiating between malignant and benign ascites, and improvement in pathologists' diagnostic accuracy
Nagata et al. (2018) [26]	Retrospective cohort study	1,720 (973 in derivation cohort, 747 in validation cohort)	Japan	Development of prediction model for postoperative peritoneal metastasis	Colon cancer	Peritoneum	Elastic net regression techniques (statistical model)	Included as a factor in model (T0-T2, T3, T4a, T4b)	Included as a factor in model (N0, N1, N2)	Development of a prediction model with good discrimination and calibration for estimating individual risk of peritoneal recurrence
Song et al. (2022) [25]	Retrospective case-control study	1,284	China	Development of nomogram for predicting peritoneal metastasis risk	Colorectal (left colon, right colon, rectum)	Peritoneum	LASSO regression and multivariate logistic regression analysis	Different stages (T1-T4)	Different stages (N0-N2)	Nomogram with C-index, AUC, and internal validation C-index
Tsai et al. (2021) [33]	Retrospective cohort study	2,003	Taiwan	Prediction model for metachronous peritoneal carcinomatosis	Colon cancer	Peritoneal	Cox prediction models	T4a and T4b	N0, N1, N2	Development of a prediction model for metachronous peritoneal carcinomatosis (mPC) risk in T4 colon cancer patients after curative resection
Yao et al. (2023) [19]	Retrospective cohort study	16,956	USA (SEER database)	Risk model for predicting peritoneal metastasis	Colorectal cancer	Peritoneal	Logistic regression analysis	T1 to T4	N0, N1, N2	Development of a predictive model for peritoneal metastasis in colorectal cancer patients
Zhang et al. (2022) [32]	Retrospective cohort study	622	China (two different centers)	Prediction model for exploratory laparoscopy risk stratification	Colorectal cancer	Occult peritoneal metastasis	Multivariate logistic regression analysis	T1 to T4	N0, N1/N2	Development of an individualized prediction model to identify occult peritoneal metastasis status and determine optimal candidates for exploratory laparoscopy
Andrieu et al. (2022) [20]	Retrospective cohort study	9,928 patients (with 48,408 CT reports)	United States	Natural language processing (NLP) applied to CT radiology reports	Colorectal cancer	Peritoneal metastasis and multiple organs	NLP prediction model with ensemble voting model built from logistic regression, support vector machine, random forest, and extreme gradient boosting models	Various (not specifically analyzed)	Various (not specifically analyzed)	Overall survival (OS) by metastatic pattern, frequency of metastases for each organ, and comparison of AJCC TNM staging versus an alternative classification based on the number of metastatic organs.
Li et al. (2020) [31]	Retrospective study	779 (585 in training set, 194 in validation set, plus an independent validation cohort of 139 patients)	China	Clinical-radiomics model for preoperative prediction of peritoneal metastasis	Colorectal cancer	Peritoneal metastasis	Radiomics with least absolute shrinkage and selection operator (LASSO) algorithm, including features of the primary lesion and largest peripheral lymph node	Not specified	Not specified	Prediction performance of synchronous peritoneal metastasis and comparison between the clinical model, the radiomics model, and the clinical-radiomics model
Li et al. (2024) [34]	Retrospective study	217 (116 with surgical pathology, 101 confirmed through endoscopy)	China	Artificial intelligence iterative reconstruction (AIIR) algorithm for CT images	Colorectal cancer	Primarily focused on visceral peritoneum invasion and hepatic metastases	Deep-learning-based CT reconstruction algorithm (AIIR)	For 116 patients with surgical pathology: pT1=9, pT2=21, pT3=78, pT4a=8	For 116 patients with surgical pathology: pN0=68, pN1=34, pN2a=8, pN2b=6	Image quality metrics, diagnostic performance for visceral peritoneum invasion, and changes in diagnostic thinking regarding hepatic metastases
Miao et al. (2025) [35]	Retrospective cohort study	416 (internal dataset) + 119 (external test set)	China	Multi-scale Feature Fusion Network (MSFF-Net) based on ResNet18	Colorectal (including right colon, left colon, and rectum)	Peritoneal	Deep learning model (MSFF-Net) that extracted features of tumors and visceral fat from CT scans	T1-T4 tumors	N0-N3 lymph node status	Prediction accuracy of peritoneal metastasis; AUC
Zhang et al. (2024) [36]	Retrospective cohort study	220 (123 training, 41 internal validation, 56 external validation)	China	Radiomics-boosted deep learning model based on ResNet50	Colorectal (right and left colon)	Peritoneal	Radiomics-boosted deep learning model using PET/CT images	NA	NA	Prediction accuracy of synchronous peritoneal metastasis; AUCs
Mhawech-Fauceglia et al. (2020) [37]	Retrospective study	23 (10 non-invasive implants, 9 invasive implants, 4 HG peritoneal metastases)	USA and Canada	Gene expression profiling using nanostring human cancer Reference panel	Ovarian borderline serous tumors	Peritoneal implants	LASSO in Glmnet package for gene selection	NA	NA	Identification of differentially expressed genes and malignant potential prediction
Pang et al. (2023) [38]	Retrospective multicenter study	178 (88 PTB patients, 90 PC patients)	China	Machine learning model based on CT findings	Various (peritoneal carcinomatosis)	Peritoneal	Logistic regression machine learning model	NA	NA	Diagnostic accuracy of machine learning model to differentiate peritoneal tuberculosis from peritoneal carcinomatosis
Schnelldorfer et al. (2019) [39]	Retrospective observational	87 peritoneal lesions from 35 patients	USA	Evaluation of laparoscopic images of peritoneal lesions by 10 oncologic surgeons and image processing algorithms	Gastrointestinal tract (pancreatic, gastric, gallbladder)	Peritoneal	Computer-aided digital image processing and machine learning with neural network	NA	NA	Accuracy of identifying peritoneal metastases based on appearance; AUC of multivariate model of nodularity, border transition, and transparency
Schnelldorfer et al. (2024) [40]	Retrospective observational	132 patients (with 4,287 visible peritoneal lesions, including 365 biopsied lesions)	USA	Development of a deep learning system (computer-assisted staging laparoscopy - CASL)	Gastrointestinal tract (adenocarcinoma)	Peritoneal	Deep learning neural network (YOLOv5 and ensemble of ResNet18 networks)	NA	NA	AUC for CASL vs AUC for surgeons
Shi et al. (2024) [21]	Retrospective, bicentric study	302 patients (training: n=167, internal test: n=72, external test: n=63)	China	CT-based deep learning-based radiomics (DLR) model	Pancreatic ductal adenocarcinoma (PDAC)	Occult peritoneal metastases (OPM)	Deep learning radiomics (DLR) and handcrafted radiomics (HCR) with UCTransNet for segmentation and logistic regression classifier	T stage as variable (58.7% T1/T2 and 41.3% T3/T4)	N stage as variable (53.3% N0 and 46.7% others)	Combined model AUC in training, internal test, and external test cohorts
Walma et al. (2024) [24]	Retrospective, bicentric study	2,262 patients in development cohort; 663 patients in validation cohort	The Netherlands, Italy	Development of a prediction model for occult metastases	Pancreatic adenocarcinoma	Liver (61%), peritoneal (31%), or both (8%)	Multivariable logistic regression analysis based on the Akaike Information Criteria (not deep learning AI but statistical modeling)	≥T3 tumor in 15% of patients without occult metastases and 22% of patients with occult metastases in development cohort	Not specifically reported in the same format, but suspected lymph node metastases were present in 15% of patients without occult metastases and 18% of patients with occult metastases	The study developed a model to predict occult metastases with moderate discrimination in the development cohort but poor discrimination upon external validation
Wang et al. (2023) [23]	Retrospective observational	168	China	CT-based deep learning model	Multiple abdominal malignancies	Peritoneal metastasis	DenseNet121-SVM model	NA	NA	Preoperative staging of peritoneal metastasis
Xia et al. (2025) [22]	Multicenter cohort	522	China	Machine learning models	Liver (ruptured HCC)	Peritoneal metastasis	Deep learning, logistic regression, SVM, classification trees, and random forests	NA	NA	Prediction of postoperative peritoneal metastasis (DL model)

Quality Assessment

The methodological quality assessment using the modified RQS revealed variable performance across the 10 included AI and radiomics studies, with total scores ranging from 10 to 26 points out of a maximum of 36 points. Li et al. achieved the highest score of 26 points, followed by Miao et al. with 25 points, indicating high methodological quality and strong potential for clinical translation [34,35]. Shi et al., Wang et al., and Zhang et al. demonstrated moderate to good quality, with scores of 22, 19, and 19 points, respectively [21,23,32]. In contrast, Kim et al. and Schnelldorfer et al. showed moderate quality, with scores of 18 and 17 points, respectively [28,39]. The remaining studies scored between 10 and 11 points, indicating lower methodological quality (Table 3).

**Table 3:** RQS quality assessment table. RQS: Radiomics Quality Score

Studies	V1	V2	V3	V4	V5	V6	V7	V8	V9	V10	V11	V12	V13	V14	V15	V16	Total
Kim et al. (2024)	2	1	1	0	3	3	0	1	2	2	1	1	0	0	0	1	18
Li et al. (2024)	2	1	0	0	-3	4	0	1	2	2	1	1	0	0	0	0	11
Li et al. (2020)	2	1	0	0	3	4	1	1	2	2	1	1	1	7	0	0	26
Miao et al. (2025)	2	1	0	0	3	4	1	1	2	2	1	1	0	7	0	0	25
Pang et al. (2023)	2	1	0	0	-3	3	0	1	2	2	1	2	0	0	0	0	11
Schnelldorfer et al. (2024)	2	1	0	0	-3	2	0	1	2	2	1	2	0	0	0	0	10
Schnelldorfer et al. (2019)	2	1	0	0	3	2	1	1	2	2	1	2	0	0	0	0	17
Shi et al. (2024)	2	1	1	0	3	3	1	1	2	2	1	2	2	0	0	1	22
Wang et al. (2023)	2	1	0	0	3	2	1	1	2	2	1	2	1	0	0	1	19
Zhang et al. (2024)	2	1	0	0	3	3	1	1	2	2	1	1	1	0	0	1	19

**Figure 2:**
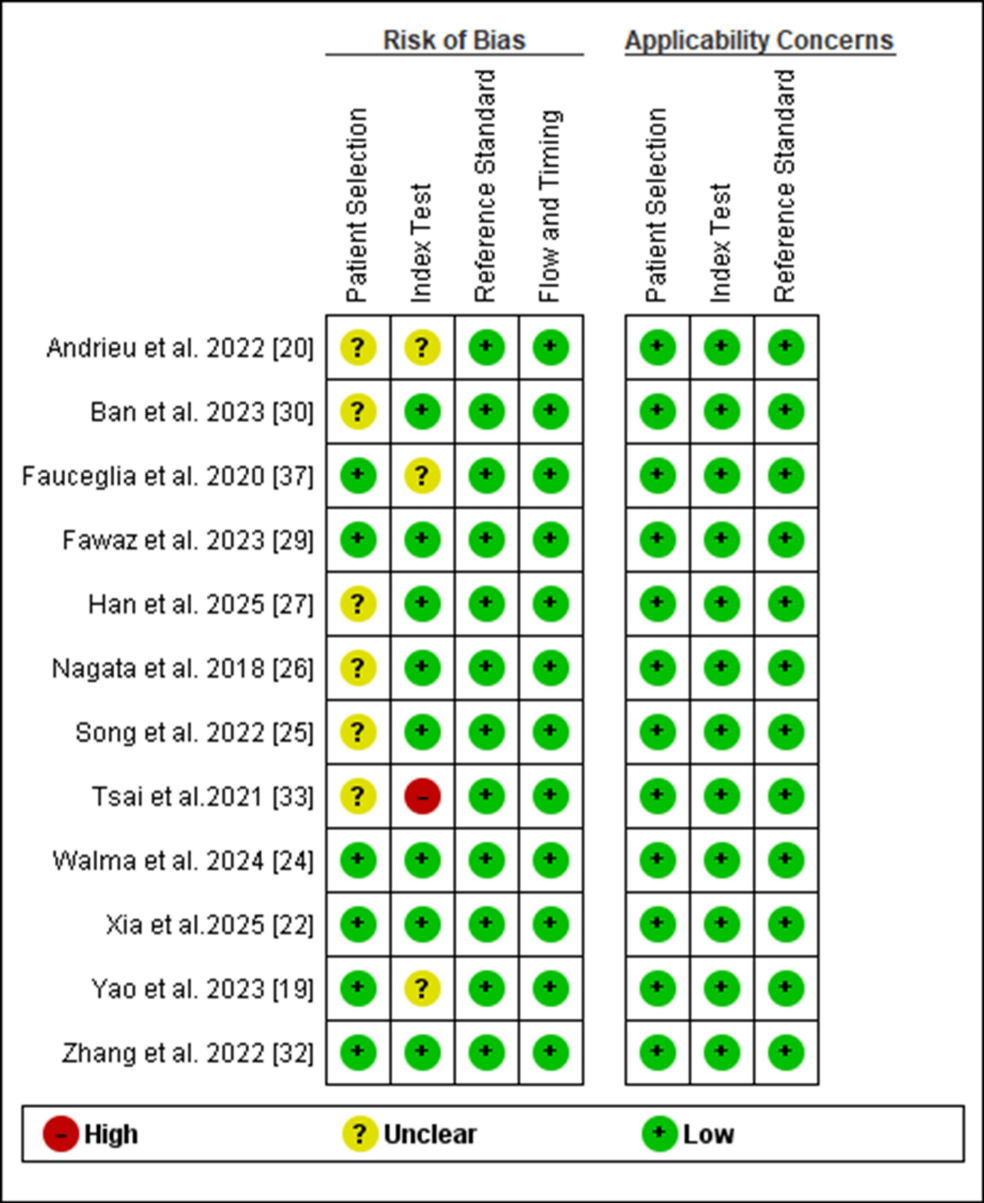
Methodological quality summary for QUADAS-2. QUADAS-2: Quality Assessment of Diagnostic Accuracy Studies-2

The QUADAS-2 quality assessment tool generally demonstrated a low risk of bias and good applicability. Most studies (10 out of 12) showed low risk of bias for patient selection, index test conduct, reference standard application, and study flow and timing, with only Andrieu et al. and Han et al. receiving unclear ratings for patient selection bias, and Tsai et al. showing high risk of bias for the index test domain [20,27,33]. Regarding applicability concerns, all studies demonstrated excellent relevance to the review question with consistently low concern ratings across patient selection, index test, and reference standard domains. The data of the summary is shown in Figure 2, and the methodological quality graph is presented in the figure in appendix.

Diagnostic Performance of AI Models

The diagnostic performance of AI models across included studies demonstrated AUC values ranging from 0.7 to 0.914 with the highest performing models achieving AUC values above 0.9 reported by Yao et al. (AUC=0.912) [19], Pang et al. (AUC=0.914) [38], and Miao et al. (AUC=0.911), while sensitivity values ranged from 0.666 to 0.937 with most studies achieving sensitivity above 0.8, specificity showed greater variation ranging from 0.55 to 0.99 with several studies demonstrating excellent specificity (>0.9) [35]. Finally, overall accuracy ranged from 0.65 to 0.937, with most studies achieving accuracy above 0.8, indicating generally good to excellent discriminative ability across different AI approaches.

AI Applications Versus Traditional Methods Performance Comparison

Traditional clinical prediction models, as demonstrated across multiple studies, consistently showed performance metrics with C-indices ranging from 0.73 to 0.85. The elastic net regression model achieved a C-index of 0.73 (95% CI: 0.68-0.78) using six clinical predictors, successfully stratifying 1,629 patients into four risk categories with five-year cumulative incidence rates ranging from 0.30% (lowest risk) to 17.8% (highest risk) [26]. Song et al.'s multi-institutional nomogram demonstrated superior discrimination, with a C-index of 0.851 (95% CI: 0.815-0.887) in the training group and 0.826 (95% CI: 0.752-0.901) in the validation group [25]. The pT4-specific model achieved exceptional accuracy with a prediction error ≤5% within the first postoperative year [33], while Zhang et al.'s occult PM detection model reached the highest traditional performance with C-indices of 0.850 (development) and 0.794 (validation), successfully identifying 76.7% of occult cases while preventing 40% of unnecessary laparoscopies at the 30% risk threshold [32]. Traditional approaches also demand extensive manual processing and are too slow for practical clinical use. And finally, CT imaging misses 89% of small cancer lesions, and pathologists' accuracy varies widely from 54% to 92% [19,28]. All results from the included studies regarding diagnostic performance are presented in Table 4.

**Table 4:** Summary of performance for the used models. NA: not available; AUC: area under the curve

Studies	AUC	Accuracy	Sensitivity	Specificity
Ban et al. (2023) [30]	0.782 (0.690-0.874)	NA	NA	NA
Fawaz et al. (2023) [29]	0.873 (0.818-0.920)	NA	0.89 (0.77-0.98)	0.73 (0.71-0.83)
Kim et al. (2024) [28]	0.8851	0.937	0.877	0.99
Han et al. (2025) [27]	0.784 (0.658-0.910)	NA	NA	NA
Nagata et al. (2018) [26]	NA	NA	NA	NA
Song et al. (2022) [25]	0.882 (0.845-0.919)	NA	NA	NA
Tsai et al. (2021) [33]	From 0.7 up to 0.8.	NA	NA	NA
Andrieu et al. (2022) [20]	NA	0.96	NA	NA
Zhang et al. (2022) [32]	0.794 (0.690-0.899)	curve	0.857	0.69
Yao et al. (2023) [19]	0.912	>0.8	NA	NA
Li et al. (2020) [31]	0.7967	NA	0.813	NA
Li et al. (2024) [34]	0.87	NA	0.75	0.94
Miao et al. (2025) [35]	0.911	0.9076	0.882 (0.803-0.898)	0.926 (0.887-0.942)
Zhang et al. (2024) [36]	0.758	0.68	0.857	0.608
Mhawech-Fauceglia et al. (2020) [37]	NA	NA	NA	NA
Pang et al. (2023) [38]	0.914	0.857	NA	NA
Schnelldorfer et al. (2024) [40]	0.78 (0.73-0.83)	0.65	0.84	0.55
Walma et al. (2024) [24]	NA	NA	NA	NA
Shi et al. (2024) [21]	0.852 (0.740-0.929)	NA	0.666	0.722
Schnelldorfer et al. (2019) [39]	0.82	0.72	NA	0.77
Xia et al. (2025) [22]	0.795	NA	NA	NA
Wang et al. (2023) [23]	0.906	0.821	0.821	0.952

On the other hand, the AI-assisted cytological detection system demonstrated superior and more consistent performance utilizing both Papanicolaou (Pap) and H&E-stained images to achieve exceptional validation performance with 95.35% accuracy, 91.17% sensitivity, and 99.52% specificity, maintaining robust test set performance at 93.74% accuracy, 87.76% sensitivity, and 99.75% specificity [28]. Miao et al. developed a multi-scale feature fusion network (MSFF-Net) that extracted features from both tumors and visceral fat, achieving an impressive AUC of 0.941 (95% CI: 0.891-0.986), which substantially outperformed traditional diagnostic methods [35]. Similarly, Zhang et al. demonstrated that their radiomics-boosted deep learning model surpassed classical radiomics approaches, achieving an AUC of 0.889 (95% CI: 0.823-0.954) [32]. The most advanced AI application combined circulating tumor DNA detection with clinical pathological factors, representing a paradigmatic shift toward precision medicine. This integrated approach identified ctDNA positivity in 59 of 299 patients (19.7%), with ctDNA-positive patients showing dramatically elevated risks as follows: 8.522-fold increased risk of peritoneal metastasis (p<0.001, OR: 8.522, 95% CI: 4.371-16.615), 32.729-fold higher risk of recurrence, and 11.244-fold higher risk of mortality compared to ctDNA-negative patients [27]. The computer-assisted staging laparoscopy (CASL) system demonstrated solid performance across both detection and classification tasks, with the detection component achieving an area under the curve-precision recall (AUC-PR) of 0.69 (95% CI: 0.66-0.72) on 1,092 images containing 4,287 lesions, while the classification system reached an area under the curve-receiver operating characteristic (AUC-ROC) of 0.78 (95% CI: 0.73-0.83) with 78% accuracy when tested on 3,650 lesion patches from 365 lesions [40]. A national survey of 111 oncologic surgeons evaluating 1,086 peritoneal lesion exams demonstrated that human diagnostic accuracy was only 52% with an AUC-ROC of 0.69, which improved significantly when combined with CASL support (AUC-ROC: 0.79) compared to CASL alone (AUC-ROC: 0.78) [37,40].

Individual staining models exhibited distinct performance characteristics. The PAP model (based on the Xception architecture) achieved 89.54% test accuracy with perfect specificity (100.00%) but lower sensitivity (78.90%), whereas the H&E model demonstrated more balanced results, with 96.67% accuracy, 93.83% sensitivity, and 99.58% specificity. The AI system processed a substantial dataset of 581 whole-slide images, generating over 70,000 patch images. The deep learning models were trained on 56,560 patches and validated on 13,602 additional patches [27,31]. Genomic heterogeneity in peritoneal implants was demonstrated through a differential analysis of gene expression using the NanoString human cancer reference panel, which identified a malignant signature [31,37].

Discussion

This systematic review of 22 studies from multiple countries examined AI applications for predicting patients with PMCC, revealing that AI-assisted approaches consistently outperformed traditional diagnostic methods across all evaluated modalities. While conventional clinical prediction models demonstrated moderate performance, traditional CT imaging failed to detect the majority of small lesions, with highly variable pathologist accuracy. In contrast, AI-powered systems demonstrated markedly superior results. AI-based cytological detection systems achieved exceptional accuracy and specificity rates of over 95%. Advanced radiomics models incorporating deep learning have significantly surpassed classical approaches, and the integration of circulating tumor DNA analysis with clinical factors has identified dramatically elevated risk levels that traditional methods could not detect. Computer-assisted staging laparoscopy has substantially improved diagnostic accuracy in humans. Overall, while AI applications show potential advantages over conventional diagnostic approaches for detecting and predicting PMCC patients, extensive validation in real-world clinical environments is essential before these technologies can be confidently integrated into routine clinical practice. However, these promising results require cautious interpretation, as most studies lack external validation across diverse clinical populations and real-world healthcare environments, limiting the generalizability of reported performance metrics.

Critical Diagnostic Challenge

The clinical imperative for PMCC prediction emerges from consistently poor prognosis across all studies; this poor prognosis represents a life-or-death diagnostic challenge. Han et al. demonstrated in their research that approximately 4-19% of people with CRC experience metastasis to the peritoneal cavity, and doctors face significant challenges in diagnosing this early, as imaging techniques have a limited ability to detect it [27]. Moreover, it is reported that when cancer spreads to the peritoneum, the outlook is quite poor; most patients live just six to nine months after being diagnosed, with more extensive spread resulting in shorter survival periods [41]. Finally, Peritoneal metastasis leads to much shorter survival times compared to cancer that spreads to other parts of the body, with patients typically living only six to eight months when left untreated [32].

Early Detection and Timely Intervention

Predicting and catching delayed PM early plays a crucial role in improving outcomes after CRC, since surgeons can more easily achieve complete cancer removal in patients who have less extensive peritoneal disease spread [30]. Early detection of peritoneal metastasis significantly impacts CRC patient outcomes since lower levels of peritoneal cancer involvement require less extensive surgery, and the amount of peritoneal disease, along with how thoroughly it can be surgically removed, are the most critical prognostic factors [32]. Han et al. found that their model effectively identifies colorectal cancer patients who are most likely to develop peritoneal metastasis after surgery, enabling doctors to detect problems sooner and intervene more quickly [27].

AI Applications Versus Traditional Methods

Traditional clinical prediction models demonstrated moderate to good performance across multiple studies, with most achieving respectable discrimination capabilities [26]. The elastic net regression approach, using clinical predictors, effectively stratified patients into distinct risk categories, showing a clear separation between low-risk and high-risk groups for developing peritoneal metastasis over time [25]. Multi-institutional nomograms showed strong discriminative ability in both training and validation cohorts, indicating good generalizability across different healthcare settings. Specialized models targeting specific patient populations, such as those with advanced tumor stages, achieved particularly high accuracy with minimal prediction errors during the critical first year after surgery [33]. The most successful traditional approaches for detecting occult PM have demonstrated excellent performance in identifying the majority of hidden cases, while significantly reducing the need for unnecessary diagnostic procedures, representing the upper limit of what conventional statistical modeling can achieve in this challenging clinical scenario [32]. However, the traditional approach still has several serious flaws. Standard clinical models struggle with complex data relationships, while advanced statistical approaches, such as elastic net, still rely on outdated linear assumptions. Traditional approaches also demand extensive manual processing and are too slow for practical clinical use [32].

Clinical Applications and Performance Validation

The prediction models serve multiple purposes throughout patient care. According to Tsai et al., doctors can quickly obtain the eight necessary predictors within a week of surgery after receiving pathology results, then use this information to guide treatment discussions with patients before starting adjuvant therapy [33]. For long-term monitoring, Ban et al. targeted their model toward identifying peritoneal metastasis that develops within three years of successful surgery [30]. Nagata et al. noted that their model utilizes clinical information that doctors can readily access in everyday practice, making it applicable across various healthcare settings [26]. Direct head-to-head comparison between AI and human pathologists revealed AI's superior consistency and diagnostic accuracy, with AI-assisted diagnosis improving pathologist performance from 73.3% to 79.3% sensitivity, 93.7% to 94.8% specificity, and 86.8% to 90.5% accuracy. At the same time, AI correctly identified eight malignant cases that all human pathologists had misdiagnosed as benign [28]. AI-enhanced approaches demonstrated superior risk stratification with the molecular AI model achieving unprecedented 8.522-fold risk amplification for ctDNA positivity while improving diagnostic sensitivity from 73.3% to 79.3%, fundamentally altering clinical decision-making through objective, reproducible assessments that eliminated inter-observer variability, and enabled personalized medicine approaches, though requiring substantial initial technology investments compared to traditional clinical models that showed more modest 2-4 fold risk ratios [27,28].

Limitations of Current Imaging Techniques

Whole-body diffusion-weighted MRI demonstrates an excellent ability to detect PMCC patients and outperforms CT scans in assessing the extent of cancer spread and measuring lesion sizes. Multiple studies have also shown that PET/CT scans perform well in identifying PM [29]. CT imaging has an 11% success rate in finding cancer lesions smaller than 0.5 cm [42]. Direct head-to-head comparison between AI and human pathologists revealed AI's superior consistency and diagnostic accuracy, with AI-assisted diagnosis improving pathologist performance from 73.3% to 79.3% sensitivity, 93.7% to 94.8% specificity, and 86.8% to 90.5% accuracy. At the same time, AI correctly identified eight malignant cases that all human pathologists had misdiagnosed as benign [28].

Real-Time Clinical Decision Support System and Benefit From Human and AI Combination

The CASL system represents a major advancement in surgical technology, providing real-time diagnostic assistance during operations, unlike traditional scans that only work before surgery. This system helps surgeons make better decisions right during the procedure when they're examining patients for peritoneal metastasis [40]. While the system's ability to find lesions needs improvement, it performs much better at determining whether found lesions are cancerous or not, often outperforming human doctors in making these classifications. The significant difference between how well doctors perform alone versus with AI assistance highlights important issues in current surgical practice that may lead to missed cancer spread and early recurrence. When surgeons utilize CASL support, their diagnostic accuracy improves significantly, demonstrating that humans and AI work more effectively together than separately. Instead of replacing surgeons, the AI system helps them by providing consistent, objective analysis that reduces human errors and subjective interpretation mistakes. The system also helps reduce unnecessary tissue samples while still effectively detecting cancer cases [39,40].

Improved Treatment Planning

Research consistently shows how predictive tools would transform surgical decision-making. According to Fawaz et al., a scoring system can help clinicians identify high-risk patients (those with scores over 168) who require more detailed imaging studies. If these specialized scans indicate possible cancer spread, surgeons could then perform diagnostic laparoscopy [29]. Zhang et al. noted that through exploratory laparoscopy, doctors could determine whether extensive cancer removal surgery would be possible based on how much disease is present and how completely it could be removed [32]. Tsai et al. found that their prediction model calculates individual patient risk and helps identify which patients with advanced colon cancer are most likely to develop peritoneal cancer spread [33]. AI-enhanced approaches demonstrate superior risk stratification capabilities, with molecular AI models achieving unprecedented risk amplification compared to traditional clinical models, which show modest 2-4 fold risk ratios. This improvement enables more precise patient selection for aggressive interventions and specialized imaging studies [27].

Current Challenges and Limitations in AI Implementation

Multiple research studies have revealed the inadequacy of current imaging methods. In today's clinical practice, multidetector CT scanning is the primary tool used to detect PM following curative surgery; however, this imaging method has poor accuracy in detecting such spread and frequently underestimates the extent of the disease present [29]. This leads to a troubling situation where surgeons discover hidden peritoneal metastasis in 10-35% of CRC patients whose CT scans had shown no signs of this cancer spread [32]. Furthermore, current AI implementations face significant workflow challenges, as demonstrated by Andrieu et al., where manual curation of radiology reports required three specialized radiologists, each given specific instructions to label for the presence or absence of metastatic disease, highlighting the labor-intensive nature that limits practical clinical adoption of current AI diagnostic systems [20]. A major obstacle in radiomics and deep learning research remains the seamless translation of research findings into clinical practice. Even advanced AI-assisted approaches require extensive manual oversight [35]. Despite promising results, current AI systems face several limitations, including dependency on large datasets, potential overfitting issues, and the need for multi-institutional validation. The moderate detection performance of some systems indicates room for improvement in identifying peritoneal lesions [40]. Another critical limitation is the lack of cost-effectiveness evaluations in the included studies. The successful translation of AI into oncology depends not only on improved diagnostic accuracy but also on rigorous external validation, integration into routine clinical workflows, and robust safety oversight, as emphasized in a recent strengths, weaknesses, opportunities, and threats (SWOT) analysis of clinical AI [43].

Strengths and Limitations of the Research

We conducted our search in a comprehensive manner to ensure the inclusion of all AI-related terminology and to gather all related articles to formulate comprehensive evidence. We conducted a dual quality assessment to ensure suitability for all included studies and to estimate their quality as accurately as possible. A multimodal approach is presented in this study, as we have included diverse AI applications, ranging from radiomics to molecular analysis, to provide a comprehensive clinical picture. The results of this study focus on clinically implementable technologies rather than purely research applications, which provide high real-world applicability and value. Finally, this study addresses well-defined diagnostic challenges with significant clinical impact to answer these questions or provide guidance for future research to focus on these challenges. Despite these previous points, this review has several limitations. Studies employed diverse ML algorithms, radiomics techniques, and deep learning architectures, making direct comparison challenging. The study didn't provide an analytical comparison between the conventional tools and deep learning or AI. According to the quality assessment, variation in methodological rigor was detected across the included studies. Several studies had limited patient cohorts, and the majority employed retrospective study designs, which may be associated with certain concerns. Finally, different studies employed varying performance metrics (AUC, sensitivity, specificity, and accuracy), which complicates the conduct of a meta-analysis and limits our research to a systematic review.

## Conclusions

This systematic review demonstrates that AI technologies offer promising advantages over traditional diagnostic methods for peritoneal metastasis detection. AI applications can support clinical decision-making and enhance surgical diagnostic accuracy, especially if made in collaboration with humans. The application of AI in hospitals can also give objective, reproducible assessments that eliminate inter-observer variability. AI can save time by analyzing complex imaging data much faster than traditional manual review. AI can detect lesions that are hard to recognize by humans and assist less experienced clinicians in decision-making.

Despite several promising results, we should consider that most studies lack external validation across diverse clinical populations and real-world healthcare environments, limiting generalizability. Additionally, current AI implementations face workflow challenges, requiring extensive manual oversight that limits practical adoption. Before widespread clinical adoption can be recommended, future research should prioritize large-scale prospective validation studies across multiple healthcare settings, external validation across diverse patient populations, and comprehensive cost-effectiveness analyses.

## Figures and Tables

**Figure 3:**
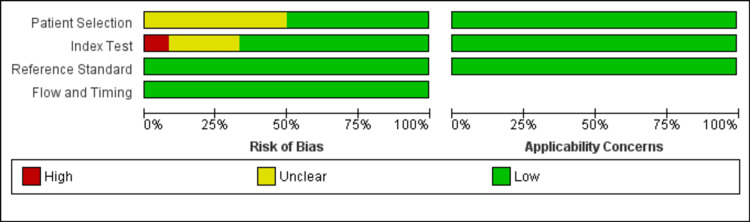
QUADAS-2 methodological quality graph. QUADAS-2: Quality Assessment of Diagnostic Accuracy Studies-2

## Appendices

Search strategy

PubMed/MEDLINE - ("artificial intelligence"[MeSH Terms] OR "artificial intelligence"[tiab] OR "computer intelligence"[tiab] OR "computational intelligence"[tiab] OR "machine intelligence"[tiab] OR "deep learning"[tiab] OR "deep neural network"[tiab] OR "machine learning"[MeSH Terms] OR "machine learning"[tiab] OR "intelligent support"[tiab] OR "intelligent agent"[tiab] OR "neural networks, computer"[MeSH Terms] OR "neural network"[tiab] OR "artificial neural network"[tiab] OR "natural language processing"[tiab] OR "nlp"[tiab] OR "decision trees"[MeSH Terms] OR "decision tree"[tiab] OR "support vector machine"[tiab] OR "reinforcement learning"[tiab] OR "supervised learning"[tiab] OR "unsupervised learning"[tiab] OR "random forest"[tiab] OR "naive bayes"[tiab] OR "k-means"[tiab] OR "convolutional neural network"[tiab] OR "gradient boosting"[tiab] OR "k-nearest neighbor"[tiab] OR "Bayesian network"[tiab] OR "principal component analysis"[tiab] OR "apriori algorithm"[tiab] OR "long-short term memory"[tiab] OR "lstm"[tiab] OR "classification model"[tiab] OR "least absolute shrinkage and selection operator"[tiab] OR "lasso"[tiab] OR "back-propagation"[tiab] OR "latent dirichlet allocation"[tiab] OR "topic-modelling"[tiab] OR "multi-layer perceptron"[tiab] OR "gated recurrent unit"[tiab] OR "max-pooling"[tiab] OR "temporal difference learning"[tiab] OR "bag-of-words"[tiab] OR "language model"[tiab] OR "word embedding"[tiab] OR "gaussian process"[tiab] OR "computer vision"[tiab] OR "fuzzy logic"[tiab] OR "expert system"[tiab] OR "expert systems"[tiab] OR "prediction model"[tiab] OR "prediction models"[tiab] OR "predictive model"[tiab] OR "predictive models"[tiab] OR "predictive algorithm"[tiab] OR "predictive algorithms"[tiab] OR "prediction algorithm"[tiab] OR "prediction algorithms"[tiab]) AND ("peritoneum"[MeSH Terms] OR "peritoneum"[tiab] OR "peritoneal"[tiab]) AND ("neoplasm metastasis"[MeSH Terms] OR "metastas*"[tiab])

Web of Science - TS=(("artificial intelligence" OR "computer intelligence" OR "computational intelligence" OR "machine intelligence" OR "deep learning" OR "deep neural network" OR "machine learning" OR "intelligent support" OR "intelligent agent" OR "neural network" OR "artificial neural network" OR "natural language processing" OR "nlp" OR "decision tree" OR "support vector machine" OR "reinforcement learning" OR "supervised learning" OR "unsupervised learning" OR "random forest" OR "naive bayes" OR "k-means" OR "convolutional neural network" OR "gradient boosting" OR "k-nearest neighbor" OR "Bayesian network" OR "principal component analysis" OR "apriori algorithm" OR "Long-short term memory" OR "lstm" OR "classification model" OR "least absolute shrinkage and selection operator" OR "lasso" OR "back-propagation" OR "latent dirichlet allocation" OR "topic-modelling" OR "multi-layer perceptron" OR "gated recurrent unit" OR "max-pooling" OR "temporal difference learning" OR "bag-of-words" OR "language model" OR "word embedding" OR "gaussian process" OR "computer vision" OR "fuzzy logic" OR "expert system*" OR "prediction model*" OR "predictive model*" OR "predictive algorithm*" OR "prediction algorithm*") AND ("peritoneum" OR "peritoneal") AND ("metastas*"))

Scopus - TITLE(("artificial intelligence" OR "computer intelligence" OR "computational intelligence" OR "machine intelligence" OR "deep learning" OR "deep neural network" OR "machine learning" OR "intelligent support" OR "intelligent agent" OR "neural network" OR "artificial neural network" OR "natural language processing" OR "nlp" OR "decision tree" OR "support vector machine" OR "reinforcement learning" OR "supervised learning" OR "unsupervised learning" OR "random forest" OR "naive bayes" OR "k-means" OR "convolutional neural network" OR "gradient boosting" OR "k-nearest neighbor" OR "Bayesian network" OR "principal component analysis" OR "apriori algorithm" OR "Long-short term memory" OR "lstm" OR "classification model" OR "least absolute shrinkage and selection operator" OR "lasso" OR "back-propagation" OR "latent dirichlet allocation" OR "topic-modelling" OR "multi-layer perceptron" OR "gated recurrent unit" OR "max-pooling" OR "temporal difference learning" OR "bag-of-words" OR "language model" OR "word embedding" OR "gaussian process" OR "computer vision" OR "fuzzy logic" OR "expert system*" OR "prediction model*" OR "predictive model*" OR "predictive algorithm*" OR "prediction algorithm*") AND ("peritoneum" OR "peritoneal") AND ("metastas*"))
